# Continuous elevation of procalcitonin in cirrhosis combined with hepatic carcinoma: a case report

**DOI:** 10.1186/s12879-020-05684-2

**Published:** 2021-01-07

**Authors:** Juan Lu, Chun-lei Chen, Jian-di Jin, Jun Chen, Cheng-bo Yu

**Affiliations:** State Key Laboratory for Diagnosis and Treatment of Infectious Diseases, National Clinical Research Center for Infectious Diseases, Collaborative Innovation Center for Diagnosis and Treatment of Infectious Diseases, The First Affiliated Hospital, College of Medicine, Zhejiang University, 79 Qingchun Road, Hangzhou, Zhejiang Province China

**Keywords:** Procalcitonin, Bacterial infection, Cirrhosis, Hepatic carcinoma, Endotoxemia

## Abstract

**Background:**

Serum levels of procalcitonin (PCT) are considered a useful biomarker for the diagnosis of bacterial infection or inflammation. There are few reports of high PCT levels in end-stage liver disease regardless of bacterial infection. Here, we present a case of extremely high PCT levels (> 100 ng/mL) in a patient with severe cirrhosis combined with hepatic carcinoma.

**Case presentation:**

A 65-year-old man developed end-stage cirrhosis with hepatic carcinoma. Radiographic imaging showed a massive hepatocellular carcinoma with multiple loci lack of indications of resection. Hence, transcatheter hepatic arterial chemoembolization was performed three times over a period of 4 months. Before and after interventional therapies, the biochemistry laboratory results were only slightly abnormal except for persistently high PCT concentrations (> 100 ng/mL), irrespective of the evidence for bacterial infection or sepsis.

**Conclusions:**

This case suggests that continuously high levels of PCT (> 100 ng/mL) may be present in advanced liver disease, particularly in complex situations such as decompensated cirrhosis and liver cancer, in the absence of severe infection or sepsis. This knowledge could expand the significance of PCT in liver disease.

**Supplementary Information:**

The online version contains supplementary material available at 10.1186/s12879-020-05684-2.

## Introduction

Procalcitonin (PCT) is a pro-peptide of calcitonin, a 13 kD glycoprotein without hormonal activity, and is secreted by thyroid C cells [1]. In healthy people under normal physiological conditions the serum concentration of PCT is so low (< 0.05 ng/mL) that it is almost undetectable [2]. PCT is also found in normal human liver, lung, kidney, and other tissues. For decades, the serum concentration of PCT has been a useful biomarker for the diagnosis of bacterial infection or inflammation [3]. In pathological states such as bacterial infections, PCT can be synthesized and secreted by the macrophages and monocytes of the liver, and the lymphocytes and endocrine cells of the lung and intestinal tissues in addition to the thyroid gland [4]. Serum levels of PCT significantly increase, remain at a high level, and gradually decrease with progression or control of the infection. However, to the best of our knowledge [2], there are very few reports of high PCT levels in end-stage liver disease without strong evidence of bacterial infection. We present here a patient with end-stage cirrhosis combined with hepatic carcinoma who had extremely high PCT levels (> 100 ng/mL), and eventually died.

## Case presentation

On September 17, 2019, a 65-year-old man was referred to our hospital with nausea and abdominal distension accompanied by fatigue and dizziness for 10 days, and a significant weight loss of 3 kg over 2 months. He had experienced chronic viral hepatitis B 20 years previously, treated with entecavir once a day. After hospitalization, physical examination revealed abdominal distension, a temperature of 37 °C, blood pressure of 125/72 mmHg, heart rate of 78 bpm, and respiratory rate of 18 bpm. Results of laboratory investigations were as follows: quantitative hepatitis B surface antigen (> 250 IU/mL) and HBV-DNA (2.97 × 10^5^ IU/mL). Routine hematological results included white blood cells (5.2 × 10^9^/L) with 68.9% neutrophils, lymphocytes (1.15 × 10^9^/L), red blood cells (4.45 × 10^12^/L), hemoglobin (129 g/L), and platelets (294 × 10^9^/L). Levels of high-sensitivity C-reactive protein (CRP) were 18.8 mg/L, and PCT level was > 100 ng/mL. Mild hepatic dysfunction was noted with an alanine aminotransferase (ALT) level of 76 IU/L, aspartate aminotransferase (AST) level of 53 IU/L, globulin (34.1 g/L), and albumin (34.6 g/L). Coagulation tests were slightly abnormal: prothrombin time (PT), 13.9 s; activated partial thromboplastin time (APPT), 33.4 s; and fibrinogen, 4.6 g/L. Renal function was essentially normal. No abnormal tumor markers were detected, except for abnormal levels of glycoproteins, at 4824 ng/ml. An initial chest CT revealed multiple subpleural nodules in the lower lobes of both lungs and the upper lobe of the right lung. Abdominal ultrasound indicated multiple hepatic masses. Contrast-enhanced CT of the whole abdomen revealed a massive left lobe hepatocellular carcinoma with multiple loci, cirrhosis, splenomegaly, and gallstones. Enhanced magnetic resonance (MRI) of the liver also showed a massive left lobe hepatocellular carcinoma with multiple loci, cirrhosis, and multiple cysts. The patient had a relatively definitive diagnosis, and was therefore treated with compound glycyrrhizin and reduced glutathione to relieve the hepatitis, via the effects on reducing enzymes and protecting hepatocytes. Entecavir was provided as an antiviral. The standard care for chronic liver disease was also administered. The patient multifocal tumors was lack of indications of resection according the practice guidelines for the management of hepatocellular carcinoma [5]. Therefore, he underwent transcatheter hepatic arterial chemoembolization (TACE) on September 23, 2019. Preoperative review of the laboratory tests showed that the PCT level had twice remained > 100 ng/mL, with no significant changes in the other indices. On the first day after surgery, the patient developed fever with a daily maximum temperature of 39.2 °C, but had no obvious discomfort other than slight fatigue. The white blood cell count was 6.2 × 10^9^/L with 94.1% neutrophils, CRP level was 28.7 mg/L, and the PCT level remained > 100 ng/mL. In view of the secondary response after the procedure and the possible absorption of necrotic materials from the carcinoma, the patient was given a non-steroidal anti-inflammatory drug, and an antibiotic (imipenem, 0.5 g three times daily). After 4 days, his temperature had decreased to 37 °C. Given the improvement in his clinical condition, the patient was discharged on September 30.

The second and third TACE procedures were performed on October 18 and December 11, 2019. Similarly, the patient developed fever with a daily maximum temperature of 39 °C approximately 1 day after each surgery. During the second hospitalization, the white blood cell count was 6.1 × 10^9^/L with 87.1% neutrophils, and the PCT level remained > 100 ng/mL on the first day after TACE. Ferritin level was 1511 ng/mL, and glycoprotein levels were abnormal, at 2455 ng/mL. The patient was not obviously uncomfortable, and he was treated with non-steroidal anti-inflammatory drugs. His temperature decreased to 37.8 °C before discharge from the hospital. During the third hospitalization, the white blood cell count was 6.9 × 10^9^/L with 90.7% neutrophils, and PCT level remained > 100 ng/mL on the first day after TACE. Ferritin level was 835 ng/mL. The patient had no obvious discomfort and was again given non-steroidal anti-inflammatory drugs. During the three hospitalizations, the patient underwent several contrast-enhanced CTs or MRIs to estimate the tumor extent or progression. The radiographic imaging always indicated giant multiple tumors, despite treatment. Lung CT scan was also underwent for differential diagnosis between benign and malignant pulmonary nodules. The application of radiological feature analysis may be particularly suited to the assessment and management of pulmonary nodules [6, 7]. Benign nodules usually smooth, round and poor growth. The size and distribution of nodules of lung metastatic tumor were not uniform density, lobulated, sometimes rough edges or ground-glass opacity [8]. The size developed rapidly and the number of nodules increased [9]. In this case, the nodules were smooth and round without lobulated. The size, density and morphological features of the major nodules in the upper lobe of the right lung remained stable (Sup 1) without systemic treatment, indicating no obvious changes during the several months.

**Fig. 1:**
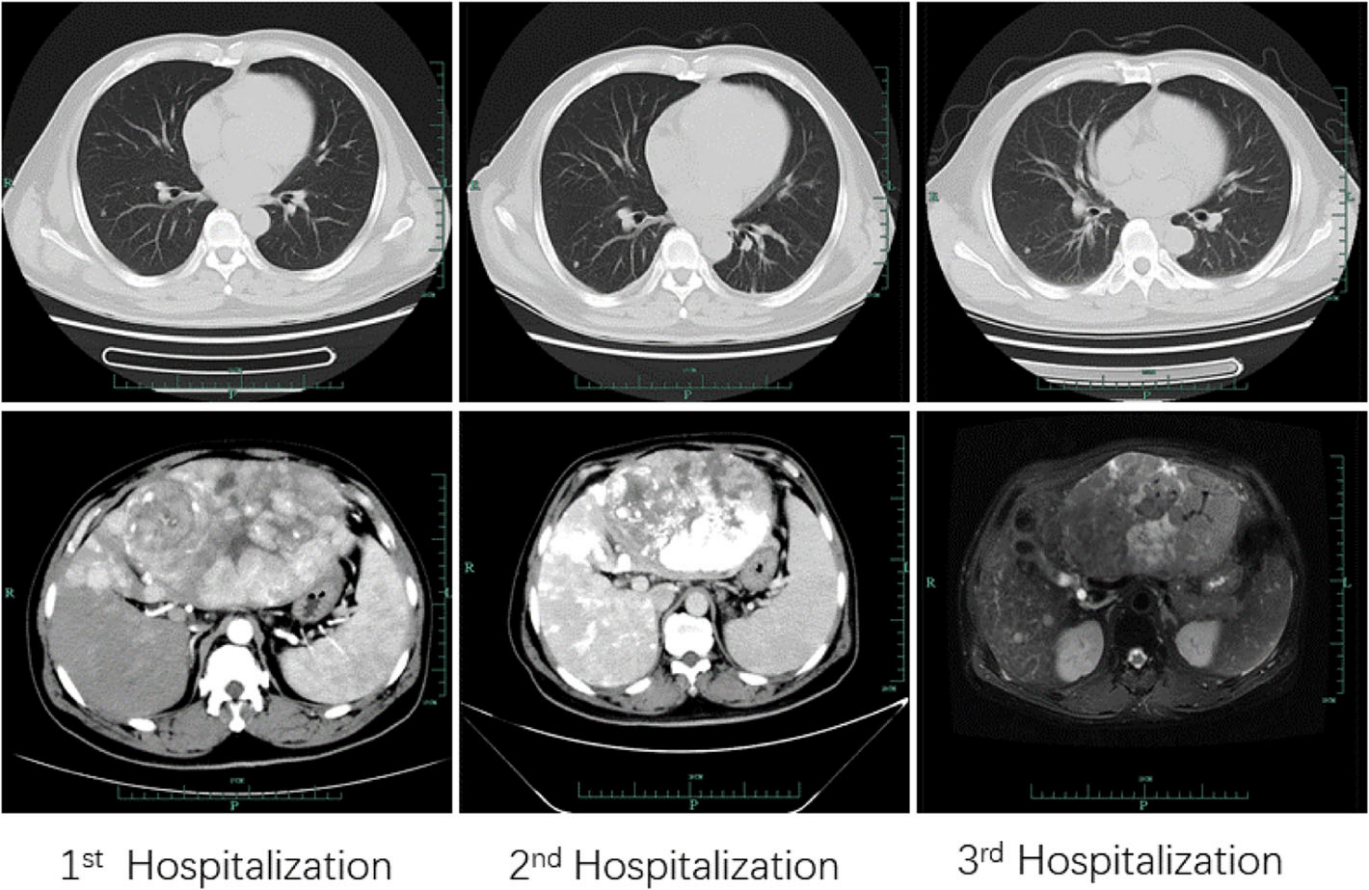
Imageological examination of three times of the hospitalization. The first row of chest CT were taken on September 19th, October 16th, December 2nd, respectively. The second row of liver CT or MRI were taken on September 20th, October 23th, December 2nd, respectively. The chest CT showed multiple subpleural nodules in lower lobe of both lungs. The enhanced CT or MRI showed the formation of left massive hepatocellular carcinoma before or after TACE therapies

The patient had no further follow-up visits after the end of 2019. When we called the patient’s family at the beginning of February 2020, his wife confirmed that the patient had died at a local hospital due to severe complications of his advanced malignancy and end-stage hepatic cirrhosis (Fig. 1).

## Discussion

It was well-known that PCT was always considered as a maker to distinguish bacterial from non-bacterial reactions [10]. However, PCT levels may be elevated in patients who do not have sepsis or infection. In some cases, PCT levels may increase significantly in certain conditions, e.g. following liver transplantation [11], during severe and prolonged cardiogenic shock [12], severe pancreatitis [13], certain types of autoimmune disorders [14] and secretory tumor such as C-cell carcinoma [15]. However, the diagnostic values and accuracy of PCT in chronic liver diseases and liver cirrhosis has been acceptable in recent findings [16]. The aim of this case was to enlighten prospective readers that high levels of PCT could be presented on end stage of tumor diseases such as in cirrhosis combined with hepatic carcinoma. Knowledge of this field is positive to enable expand the significance of PCT in liver diseases.

As this case demonstrated, we represented PCT of continuous more than 100 ng/ml on a patient with advanced liver cancer for more than 4 months. We also monitor body temperature and physical condition, dynamically observe neutrophils, leukocyte, PCT, CRP levels, blood culture and administer three times of TACE during the hospitalization. Although obvious fever after three times of TACE, he remained favorable conditions with no related complaints after the treatment of non-steroid antiinflammatory drugs. We considered that these abnormity was interrelated to secondary response after TACE. Moreover, slightly increased CRP levels (Sup 2) and slightly increased neutrophils in leukocyte (Sup 3) were detected, while the levels of neutrophils and leukocyte almost kept within normal range. It was well-known that spontaneous bacterial peritonitis is one of the most recognized clinical manifestations of bacterial translocation in chronic liver diseases and cirrhosis [17]. This patient have unobvious evidences of symptoms and signs, e.g. abdominal distention or pain and ascites. As a result, bacterial evidence was always insufficient yet during the admission of the patient to hospital.

Various tissues could produce PCT, however, the liver is still the most crucial site of PCT production [18]. In liver diseases, PCT determination was referred to be associated with disease severity combined with liver cirrhosis and to be evaluated the prognoses, in spite of the presence or absence of bacterial infections, which suggested an intricate relationship between liver and PCT [2]. In liver advanced diseases, the liver’s capability to eliminate toxins and metabolites is sharp decline, endotoxemia suffering from damage to the intestinal mucosal barrier would lead to an increase in serum concentrations of PCT [19]. Hepatocyte damage and intestinal permeability were noted to be increased in cirrhosis and also associated with endotoxemia [20–22]. The defense mechanisms of filtration and detoxication were impaired in the cirrhotic liver, finally resulting in spillover of products and secretion from various mediators. Sterile inflammation by circulating endotoxin inducing immune dysfunction may have some effect via noninflammatory mediators such as vasoactive substances [23]. Depressed elimination of endotoxin is considered to induce spillover endotoxemia which secrete larger amount of tumor necrosis factors [12]. These mechanism could be account for the increased PCT concentrations in patients with advanced liver diseases, such as decompensated liver cirrhosis in this case.

PCT determination has also expanded to other fields, such as in some neoplastic situations. Some solid tumors (thyroid carcinoma) as well as some hematological malignancies are thought to be associated with PCT positivity [24, 25]. Due to PCT production from the C-cell of thyroid and in neuroendocrine cells, spontaneous PCT increase in neuroendocrine cells may be detected. Some studies illustrated that PCT could be better discriminate infections and para-neoplastic fever [15, 24]. Liver cancer may affect various endogenetic hormone levels in vivo because liver was also an endocrine organ. Therefore, it could be easily understand that endocrine cancers are associated with PCT increasing [26].

During the management of patients with high PCT levels, the most important of all, identifying infection or non-infection is the first step in the diagnosis of patients who have ambiguous bacterial infection. Once infection was suspected, empirical antimicrobial treatment could be arranged following the clinical guideline including infection source, site of infection onset and individual risk factors for multidrug resistant infections [27]. Especially, the double infection or atypical infection should be recognized according to the clinical symptoms and signs and related findings. However, in the event of absence of infection, such as the case in this study, antibiotics should be used with cautions. Mallet suggest that the diagnostic value of PCT in patients with liver failure is related to the cause of disease [28]. As a result, treatment for primary diseases and drugs to rescue the principle of symptomatic treatment, such as non-steroidal anti-inflammatory drugs (NSAIDs) could be adopted, owing to the relative safety and relief of the symptoms [29].

## Conclusion

In conclusion, our case suggested that continuous high-level PCT concentrations (> 100 ng/ml) could be present in advanced liver diseases, especially in complex complications such as decompensated cirrhosis and liver cancer, in the absence of serve infections or sepsis. Knowledge of this field is positive to enable expand the significance of PCT in liver diseases.

## Supplementary Information

**Additional file 1: Sup 1.** The sizes and densities of nodules were computed in chest CT scans during the three hospitalizations (September 19, October 16, and December 2, 2019, respectively). The first row showed the radius (0.30, 0.30 and 0.27 cm, respectively) and area (0.28, 0.28, and 0.24 square centimeter, respectively) of the major nodules in the right lung. The second row showed the relative densities (− 406.666667, − 376.666667, − 378.500000, respectively, compared to the density of water) of the nodules.

**Additional file 2: Sup 2.** The levels of PCT and CRP during the three hospitalizations.

**Additional file 3: Sup 3.** The levels of white blood cells, neutrophils, proportion of neutrophils during the three hospitalizations.

## Data Availability

All data analyzed during this study are included in this published article.

## Abbreviations

PCT: Procalcitonin
CRP: C-reactive protein
ALT: Alanine aminotransferase
AST: Aspartate aminotransferase
PT: Prothrombin time
APTT: Activated partial thromboplastin time
MRI: Magnetic resonance
TACE: Transcatheter hepatic arterial chemoembolization
