# Supplementary material for: Continuous elevation of procalcitonin in cirrhosis combined with hepatic carcinoma: a case report
Source: BMC Infect Dis. 2021 Jan 7;21:29. doi: 10.1186/s12879-020-05684-2 (PMC7792198; doi:10.1186/s12879-020-05684-2)
Supplement: Supplementary file 1 — Additional file 1: Sup 1. The sizes and densities of nodules were computed in chest CT scans during the three hospitalizations (September 19, October 16, and December 2, 2019, respectively). The first row showed the radius (0.30, 0.30 and 0.27 cm, respectively) and area (0.28, 0.28, and 0.24 square centimeter, respectively) of the major nodules in the right lung. The second row showed the relative densities (− 406.666667, − 376.666667, − 378.500000, respectively, compared to the density of water) of the nodules. [file 12879_2020_5684_MOESM1_ESM.docx]

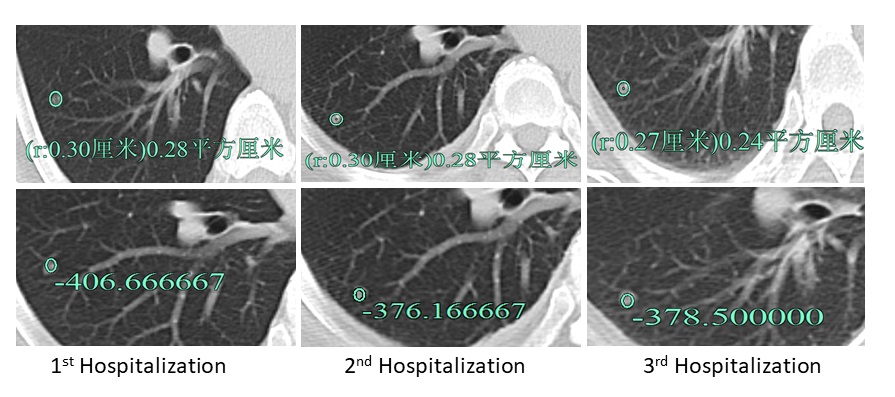


Sup 1. The sizes and densities of nodules were computed in chest CT scans during the three hospitalizations (September 19, October 16, and December 2, 2019, respectively). The first row showed the radius (0.30, 0.30 and 0.27cm, respectively) and area (0.28, 0.28, and 0.24 square centimeter, respectively) of the major nodules in the right lung. The second row showed the relative densities (-406.666667, -376.666667, -378.500000, respectively, compared to the density of water) of the nodules.
