# Supplementary figures and images for: Continuous elevation of procalcitonin in cirrhosis combined with hepatic carcinoma: a case report
Source: BMC Infect Dis. 2021 Jan 7;21:29. doi: 10.1186/s12879-020-05684-2 (PMC7792198; doi:10.1186/s12879-020-05684-2)

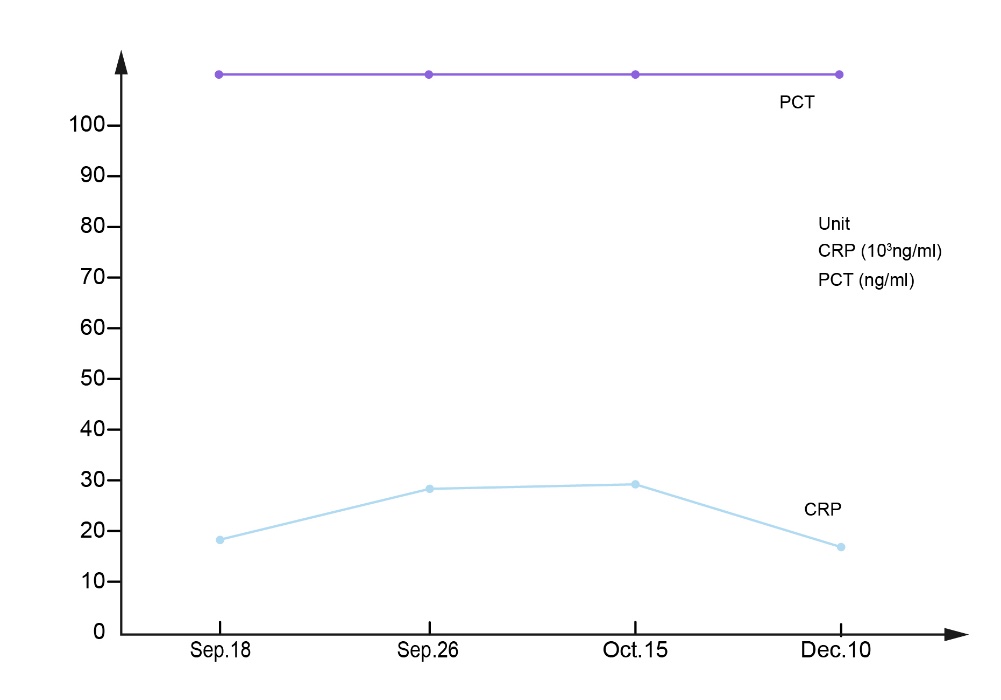


Sup 2. The levels of PCT and CRP during the three hospitalizations.

Supplement: Supplementary file 2 — Additional file 2: Sup 2. The levels of PCT and CRP during the three hospitalizations. [file 12879_2020_5684_MOESM2_ESM.docx]
