# Supplementary material for: Continuous elevation of procalcitonin in cirrhosis combined with hepatic carcinoma: a case report
Source: BMC Infect Dis. 2021 Jan 7;21:29. doi: 10.1186/s12879-020-05684-2 (PMC7792198; doi:10.1186/s12879-020-05684-2)
Supplement: Supplementary file 3 — Additional file 3: Sup 3. The levels of white blood cells, neutrophils, proportion of neutrophils during the three hospitalizations. [file 12879_2020_5684_MOESM3_ESM.docx]

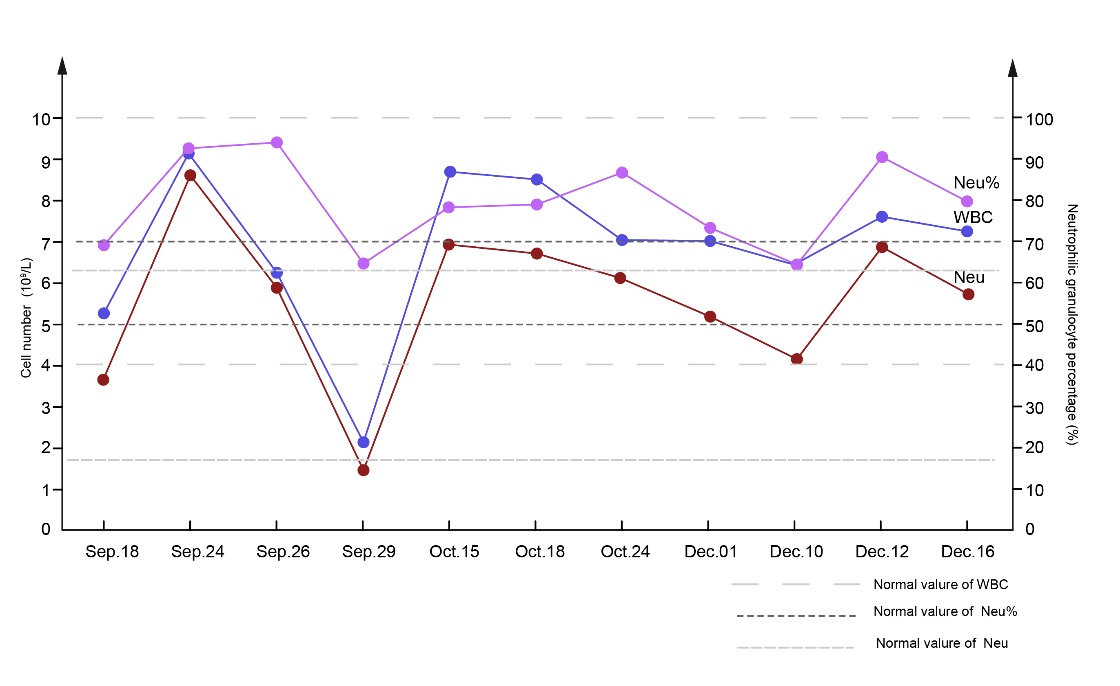


Sup 3. The levels of white blood cells, neutrophils, proportion of neutrophils during the three hospitalizations.
